# Trends in the global burden of paediatric lower respiratory infections

**DOI:** 10.1016/S1473-3099(19)30557-2

**Published:** 2019-10-31

**Authors:** Carina King, Eric D McCollum

**Affiliations:** Department of Public Health Sciences, Karolinska Institutet, Stockholm, Sweden (CK); Institute for Global Health, University College London, London WC1N 1EH, UK (CK); Eudowood Division of Pediatric Respiratory Sciences, Department of Pediatrics, Johns Hopkins School of Medicine, Baltimore, MD, USA (EDMcC); and Department of International Health, Johns Hopkins Bloomberg School of Public Health, Baltimore, MD, USA (EDMcC)

As the leading cause of infectious deaths in children,^1^ reducing lower respiratory infections (LRIs) will be the key to achieving Sustainable Development Goal 3.2. However, of the biggest infectious killers, LRI remains the condition with the least funding on a global level.^2^ In *The Lancet Infectious Diseases*, the GBD 2017 Lower Respiratory Infections Collaborators^3^ present modelled trends in LRI incident cases and mortality and estimate the role of 13 modifiable protective and preventive risk factors in these spatiotemporal changes using the Global Burden of Disease (GBD) dataset.

They^3^ report considerable reductions in LRI indicators over the last 30 years, with a 67·2% (95% uncertainty interval [UI] 63·5–70·1) reduction in LRI mortality rate and a 32·4% (27·2–37·5) reduction in incidence among children younger than 5 years. Key factors in these reductions have been the roll-out and scale-up of vaccine coverage against *Haemophilus influenzae* type b (Hib) and pneumococcal infections (pneumococcal conjugate vaccine [PCV]), improved childhood nutrition and feeding practices, and declines in household air pollution. The impressive scale of the analysis should be commended for filling a vital gap in benchmarking the role of preventive and protective interventions across broad regions.

To translate these estimates into action, intensified commitments are needed to improve the availability of high-quality local epidemiological data on both risks and causes of LRIs—especially how these vary within smaller, subnational regions. Li and colleagues^4^ have highlighted the importance of global seasonal variation in the circulation of common viral pneumonia pathogens, with considerable within-country differences. This finding raises important questions about national and regional LRI prevention programming and suggests that more nuanced approaches to vaccination strategies could achieve improved control. With more granular data on the risk factors associated with pneumonia, the same variability would probably be observed in the role and implementation of different prevention and protection interventions for paediatric pneumonia.

The two countries with the highest estimated numbers of under-5 LRI deaths—India (185 429 deaths [95% UI 167 676–204 328]) and Nigeria (153 069 deaths [115 332–196 193]), which account for 41·8% of the global under-5 LRI mortality (808 920 deaths [747 286–873 591])—serve as important examples. According to the GBD 2017 Lower Respiratory Infections Collaborators,^3^ improved nutrition-related factors and reductions in household air pollution were the main contributors to impressive reductions in mortality in India. However, absolute differences and change in rates of stunting, wasting, and underweight across India have been heterogenous.^5,6^ In Nigeria, low birthweight and short gestation was estimated to have increased LRI mortality in between 1990 and 2017, whereas Hib coverage was the third largest contributor to LRI mortality reduction. A 2016 study reported that the odds of low birthweight was 10·6 times higher in the northwest than in the southwest of Nigeria,^7^ and the 2018 Demographic and Health Survey, which gathered data from national household surveys, report vaccine coverage to range from 73% for PCV and 74% for Hib in Abuja state to 6% for PCV and 7% for Hib in Sokoto state.^8^

These estimates highlight the need for caution in extrapolating broader conclusions on the roles of different interventions and the public health gains made across heterogeneous countries with little subnational data. To make meaningful, sustainable, and equitable gains, the global health community needs to be mindful of the complex nature of LRI as a disease of poverty and consider the interface between more individualised risks and the wider determinants for health at the community, health-system, and policy levels. For example, the GBD 2017 Lower Respiratory Infections Collaborators^3^ present an interesting finding on the role of air pollution. With rapid urbanisation and economic development, reductions in household air pollution occur simultaneously with increasing ambient air pollution outside the home. To date, trials targeted at reducing indoor air pollution in low-income rural settings at a household-level have largely been ineffective,^9^ although results of the HAPIN trial (ClinicalTrials.gov, NCT02944682) in Rwanda, Peru, Guatemala, and India have not been published yet. Evidence would currently suggest that meso-level or macro-level approaches are needed; however, whether these interventions could be done in a manner that results in equitable reductions in LRI morbidity and mortality is unclear.

The GBD 2017 Lower Respiratory Infections Collaborators’ Article^3^ is an important step in quantifying broader priority areas for the prevention of paediatric pneumonia and in highlighting areas in which successes have been achieved. However, the development of local capacity to collect, analyse, and report on LRI-specific indicators needs to be intensified to ensure high-quality data for stakeholders to act effectively. We hope this work will further galvanise and build on global commitments to accelerate progress towards LRI morbidity and mortality targets and encourage funding to establish sustainable data structures and rigorously evaluate equitable effects of interventions both between and within regions.

## References

[1] Liu L, Oza S, Hogan D, Global, regional, and national causes of unde0070032–5 mortality in 2000–15: an updated systematic analysis with implications for the Sustainable Development Goals. Lancet 2016; 388: 3027–35. 27839855 10.1016/S0140-6736(16)31593-8 PMC5161777

[2] Brown R, Head M. Sizing up pneumonia research: assessing global investments in pneumonia research 2000–2015. Southampton: University of Southampton, 2018.

[3] GBD 2017 Lower Respiratory Infections Collaborators. Quantifying risks and interventions that have affected the burden of lower respiratory infections among children younger than 5 years: an analysis for the Global Burden of Disease Study 2017. Lancet Infect Dis 2019; published online Oct 30. 10.1016/S1473-3099(19)30410-4. PMC7185492 31678026

[4] Li Y, Reeves RM, Wang X, Global patterns in monthly activity of influenza virus, respiratory syncytial virus, parainfluenza virus, and metapneumovirus: a systematic analysis. Lancet Glob Health 2019; 7: e1031–45. 31303294 10.1016/S2214-109X(19)30264-5

[5] Menon P, Nguyen PH, Mani S, Kohli N, Avula R, Tran LM. Trends in nutrition outcomes, determinants, and interventions in India (2006–2016) New Delhi: Poshan, 2017.

[6] Masoud S, Menon P, Bhutta ZA. Addressing child malnutrition in India. In: Preedy VR, Patel VB, eds. Handbook of famine, starvation, and nutrient deprivation: from biology to policy. Cham: Springer, 2019: 93–108.

[7] Dahlui M, Azahar N, Oche OM, Aziz NA. Risk factors for low birth weight in Nigeria: evidence from the 2013 Nigeria Demographic and Health Survey. Glob Health Action 2016; 9: 28822. 26790460 10.3402/gha.v9.28822 PMC4720686

[8] National Population Commission. Nigeria demographic and health survey 2018—key indicators report. Abuja, and Rockville, MD: National Population Commission and ICF, 2019.

[9] Mortimer K, Balmes JR. Cookstove trials and tribulations: what is needed to decrease the burden of household air pollution? Ann Am Thorac Soc 2018; 15: 539–41. 29466681 10.1513/AnnalsATS.201710-831GH

